# Renal oncocytoma: Α case report and literature review

**DOI:** 10.18632/oncotarget.28893

**Published:** 2026-07-02

**Authors:** Areti Kalfoutzou, Cleopatra Rapti, Vasiliki Lagopulou, Eleftheria Bagiokou, Margaritis Tsantopoulos, Nikolaos Chaleplidis, Vasileios Ramfidis

**Affiliations:** ^1^Second Propaedeutic Department of Internal Medicine, Attikon General Hospital, National and Kapodistrian University of Athens, Athens, Greece; ^2^Department of Medical Oncology, 251 Air Force General Hospital, Athens, Greece; ^3^Department of Pathology, 251 Air Force General Hospital, Athens, Greece; ^4^Oncology Unit, 3rd Department of Internal Medicine, Athens General Hospital of Thoracic Diseases “Sotiria”, National and Kapodistrian University of Athens, Athens, Greece

**Keywords:** cryoablation, image-guided biopsy, kidney neoplasms, minimally invasive surgery, renal oncocytoma

## Abstract

Renal oncocytoma is a rare benign kidney tumor arising from the intercalated cells of the distal renal tubules. These tumors are often asymptomatic and incidentally discovered on imaging. This report presents an 82-year-old female patient with gross hematuria, who was subsequently diagnosed with a small renal oncocytoma. A computed tomography (CT) scan of the abdomen revealed a lesion in the upper left kidney, diagnosed as a renal oncocytoma by histopathological examination of a tissue specimen obtained via a CT-guided biopsy. After multidisciplinary review, the tumor board recommended local treatment with percutaneous cryoablation due to the lesion’s small size and indolent nature. The patient has been on annual follow-up with imaging showing no disease progression over two years. This case highlights the management considerations for renal oncocytoma, particularly in elderly patients, and underscores the role of biopsy and multidisciplinary evaluation in deciding optimal, minimally invasive treatment strategies for benign renal tumors.

## INTRODUCTION

Renal oncocytoma is a rare neoplasm, accounting for 5–9% of all renal epithelial tumors [1]. While often asymptomatic and discovered incidentally on imaging, oncocytomas may present with symptoms such as hematuria or flank pain [2]. Imaging techniques, including CT and MRI scans, aid in locating the lesion but are not able to differentiate oncocytomas from other renal tumors. Histopathological and immunohistochemical examination remains essential for diagnosis, with percutaneous biopsy increasingly accepted as a safe, minimally invasive diagnostic method [3]. In cases where the tumor is small and asymptomatic, a conservative approach, including active surveillance or minimally invasive ablation may be suitable [4]. This report discusses the case of an elderly female patient with a small renal oncocytoma managed by ablation, emphasizing diagnostic challenges and management strategies.

## CASE PRESENTATION

An 82-year-old female, non-smoker, was referred for episodes of gross hematuria for the past two months. Her past medical history was significant for diabetes mellitus, arterial hypertension and depressive disorder. She did not report any alcohol or illicit drug use. The clinical examination was unremarkable, with bilateral negative Giordano signs. Laboratory examinations were within normal limits, apart from a urine dipstick that revealed hematuria (+++). A CT scan of the abdomen with intravenous contrast demonstrated an exophytic hypodense lesion measuring 55 × 34 mm, arising from the upper left kidney (Figure 1). Based on imaging characteristics, the lesion was consistent with a Bosniak category IV renal mass, suggestive of a malignant neoplasm. Staging CT scans of the brain and chest were negative for signs of metastatic disease.

**Figure 1:**
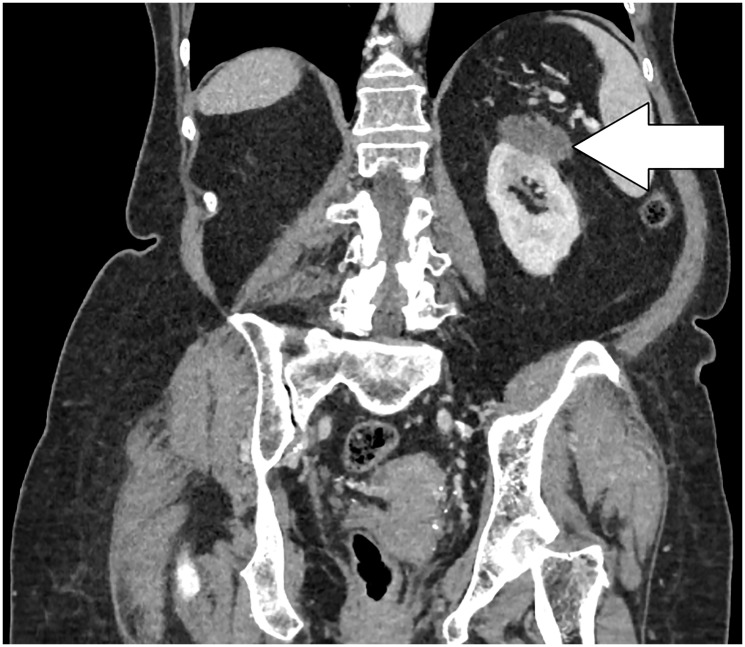
Coronal CT scan of the abdomen with intravenous contrast demonstrating a hypodense lesion measuring 55 × 34 mm arising from the upper of the left kidney.

A CT-guided percutaneous biopsy was performed, and microscopic examination showed cells characterised by nuclear atypia, infiltrating deeply into the muscle tissue and loose fibrous tissue (Figure 2A). Immunohistochemically, the tumor cells stained positive for E-cadherin (Figure 2B) and cyclin D1 and focally positive for cytokeratin 7 (CK7) (Figure 3A), and negative for CD117 (Figure 3B), Carbonic Anhydrase IX (CAIX), vimentin and RCC. These features were consistent with a renal oncocytoma. The case was discussed on a Multidisciplinary Tumor Board, and local treatment with percutaneous cryoablation was decided due to the small size and the indolent nature of the tumor. The patient is currently under annual follow-up with serial MRI scans of the abdomen and CT scans of the chest and brain and is free of disease progression for more than 2 years.

**Figure 2:**
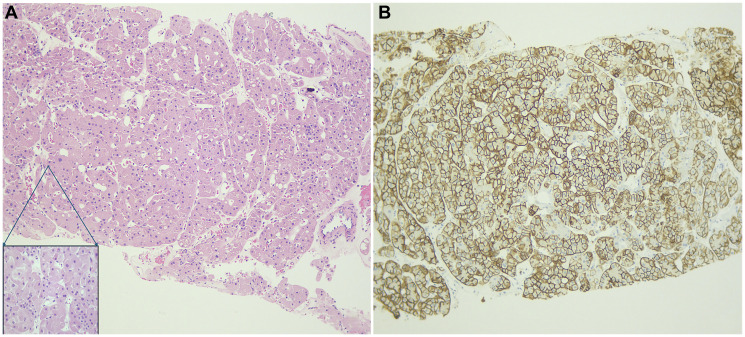
Histopathologic examination demonstrates cells with distinct cell borders, eosinophilic cytoplasm, and uniformly small-sized nuclei, without perinuclear clear halo, significant atypia, or an increased number of mitoses (hematoxylin–eosin, ×10 magnification; (**A**)). A boxed inset in the lower left of panel A highlights the tumor cells at higher magnification (×40). The cells stain positive for E-cadherin (×10 magnification; (**B**)).

**Figure 3:**
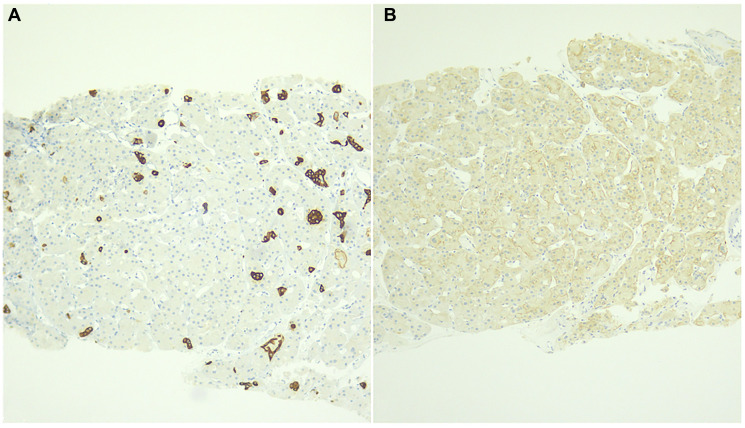
Immunohistochemical examination (X10 magnification) reveals focal positive staining for CK7 (**A**) and negative staining for CD117 (**B**).

## DISCUSSION

Renal oncocytoma is a benign tumor originating from intercalated epithelial cells of the distal collecting ducts of the kidneys [2]. It has an age-standardized incidence of 0.3 per 100.000 cases and usually affects adults during the 6th–7th decade of life, with a 2-3:1 male predominance [5, 6]. An earlier age at diagnosis or multifocal tumor location is often associated with hereditary syndromes such as Birt-Hogg-Dubé or Von Hippel-Lindau and chromosomal anomalies, including loss of 1 and Y chromosomes [1]. The majority of cases are asymptomatic and are diagnosed incidentally during imaging scans performed for other reasons [7]. In symptomatic patients, flank or abdominal pain and gross hematuria are the primary manifestations [2].

Imaging scans such as CT or Magnetic Resonance Imaging (MRI) scans are useful for locating the tumor, but are rarely able to differentiate from RCC, chromophobe RCC, papillary RCC or renal metastases [4]. On CT scan, oncocytoma usually appears as an exophytic small lesion, typically measuring less than 4 cm [5]. On MRI, oncocytoma exhibits a low signal intensity on T1 and a high signal intensity on T2-weighed images [2]. Central scarring, commonly known as the central stellate sign, is a distinctive feature on CT or MRI scan, appearing in approximately one third of cases, although it is not a unique indicator of oncocytoma [7]. Moreover, Fluorodeoxyglucose Positron Emission Tomography (FDG PET) is not an accurate modality for diagnosing renal lesions [5]. In our case, the tumor appeared as an exophytic hypodense lesion on CT scan, without central scarring.

Diagnosis relies on histopathological examination from a tissue specimen typically obtained via complete surgical excision or a percutaneous biopsy, which is gaining increasing acceptance after demonstrating safety with a low complication rate in large cohorts [3]. However, in cases of suspected RCC complete surgical excision remains the gold standard [8]. Microscopically, the tumor consists of large, round or polygonal cells with a granular eosinophilic cytoplasm, embedded within a loose edematous connective tissue stroma [1, 9]. Immunohistochemical examination usually reveals positive staining for CD117 and negative staining for CAIX and vimentin [10]. Staining for CK7, which is usually negative or focally positive in renal oncocytoma and positive in chromophobe RCC, can aid in distinguishing between these two tumor types [5]. Interestingly, tumor cells in our case stained negative for CD117. Additionally, cyclin D1 has emerged as a useful discriminatory marker, showing strong nuclear positivity in most renal oncocytomas and negativity in chromophobe RCC [11]. Cytogenetically, renal oncocytomas typically demonstrate loss of chromosomes 1 and Y, with otherwise limited chromosomal aberrations [1]. In contrast, chromophobe RCC is characterized by multiple chromosomal losses, often involving chromosomes 1, 2, 6, 10, 13, 17, and 21, reflecting a far more complex genomic background [12]. Distinction of renal oncocytoma from hybrid oncocytic/chromophobe tumor (HOCT) is another diagnostic challenge for the pathologist. HOCTs show combined features of renal oncocytoma and chromophobe RCC and are frequently associated with Birt–Hogg–Dubé syndrome or multifocal oncocytic lesions. Histologically, they often demonstrate an admixture of classic oncocytic areas with regions resembling chromophobe RCC [12]. Immunohistochemically, HOCTs typically show broader and more diffuse CK7 expression than pure oncocytomas, while retaining some oncocytic features [13]. Genomically, they tend to harbor more complex chromosomal abnormalities than oncocytoma—though still fewer and less widespread than those characteristic of chromophobe RCC [14]. In our case, the absence of diffuse CK7 staining and the lack of morphologic features indicative of chromophobe differentiation supported the diagnosis of renal oncocytoma.

Prognosis is generally excellent due to the tumor’s lack of malignant potential [5]. Perinephric fat and vascular invasion, occurring in about 8 and 6% of cases respectively, were considered as bad prognosticators, but recent evidence suggests that they do not affect patient outcome [1, 9]. A watchful waiting or active surveillance approach is appropriate for small asymptomatic tumors, whereas an open or laparoscopic surgical excision, preferably with a nephron-sparing approach if feasible, is indicated in large, growing or symptomatic tumors [4, 8]. Percutaneous cryoablation offers a non-surgical alternative to select cases not amenable to surgery [5]. Overall, careful management tailored to the tumor’s size, growth pattern, and symptomatology can provide excellent outcomes for patients while preserving renal function.

## CONCLUSIONS

This case report illustrates the management of renal oncocytoma in an elderly patient, underscoring the importance of a multidisciplinary approach in treatment planning. For small, asymptomatic tumors in elderly or high-risk patients, minimally invasive options such as cryoablation can offer effective disease control with reduced morbidity compared to surgical resection. Although imaging aids in tumor localization, histopathological confirmation via biopsy is essential for accurate diagnosis. This case supports the strategy of individualized treatment for renal oncocytoma, balancing tumor characteristics, patient comorbidities, and potential treatment risks to achieve optimal outcomes.
